# Sleep quality and response after rotator cuff repair, total shoulder arthroplasty, and reverse shoulder arthroplasty

**DOI:** 10.1016/j.jseint.2025.05.034

**Published:** 2025-06-14

**Authors:** Evan A. O'Donnell, Sally Jo, Florian Grubhofer, Jillian E. Haberli, Nicholas Wiley, Lukas Ernstbrunner, Jon J.P. Warner

**Affiliations:** a Department of Orthopaedic Surgery, Massachusetts General Hospital, Boston, MA, USA; b Department of Orthopaedics, Balgrist University Hospital, University of Zurich, Zurich, Switzerland

**Keywords:** Shoulder, Rotator cuff, Shoulder arthroplasty, Reverse arthroplasty, Rotator cuff repair, Sleep quality, Sleep disturbance

## Abstract

**Background:**

Sleep disturbance secondary to shoulder pathology is a common complaint. Previous studies have shown improvement in sleep quality after common shoulder procedures. The purpose of this study was to assess sleep quality response after arthroscopic rotator cuff repair (aRCR), anatomic total shoulder arthroplasty (aTSA), and reverse total shoulder arthroplasty (rTSA). Our hypothesis was that sleep disturbance is greater in degree and longer lasting in postoperative recovery after aRCR compared to shoulder arthroplasty.

**Methods:**

The Pittsburgh Sleep Quality Index and Visual Analog Scale–Quality of Sleep were prospectively collected in consecutive patients undergoing aRCR, aTSA, and rTSA between 2018 and 2020. Sleep quality and patient reported outcome measures, including the American Shoulder and Elbow Surgeons shoulder score, Single Assessment Numeric Evaluation (SANE) score, and visual analog scale pain score, were measured preoperatively and at 2 weeks, 6 weeks, 3 months, and 6 months postoperatively. Patient demographics, preoperative diagnosis, and comorbidities were recorded. Univariate and multivariate analyses were performed. Correlations between sleep quality metrics and patient reported outcome measures were assessed.

**Results:**

One hundred forty-one patients who underwent shoulder surgery participated in this study (aRCR: n = 34, aTSA: n = 58, rTSA: n = 49). With all shoulder surgeries pooled together, there were significant improvements in sleep quality as measured by Pittsburgh Sleep Quality Index and Visual Analog Scale–Quality of Sleep from preoperative to final follow-up (8.8 vs. 6.0, 55.4 vs. 75.2, *P* < .01 for both, respectively). The rate and magnitude of sleep quality improvement varied by surgical intervention. Sleep quality after aTSA and rTSA showed statistical improvement by 6 weeks postoperatively, which was durable through final follow-up. In contrast, after aRCR patients demonstrated a trend toward worsening sleep quality at 2 weeks with improvement by 3 months postoperatively. In multivariable regression analyses, only the type of surgical intervention, and not preoperative diagnosis or comorbidities, was associated with sleep quality at the final follow-up. Quality of sleep strongly correlated with the SANE score (r = 0.45, *P* < .01).

**Conclusion:**

Sleep quality improves after shoulder surgery, although the rate of recovery varies by surgical intervention. Sleep quality improves more rapidly after shoulder arthroplasty when compared to aRCR. The SANE score may be a useful surrogate metric in assessing sleep quality.

Nocturnal shoulder pain and sleep disturbance are common in patients with rotator cuff tears, glenohumeral osteoarthritis, and rotator cuff arthropathy. These complaints are one of the primary motivators for patients to pursue surgical intervention when conservative measures fail.^15^ While medical literature portrays the profound impact of disturbed sleep on quality-of-life measures and mental and physical health, orthopedic literature on the subject remains limited.^13^

Nocturnal shoulder pain has been reported as the strongest predictor of poor sleep in patients with shoulder pathologies.^12^ The onset of night pain may be related to the circadian rhythm and increased production of melatonin at night.^10^ The joint capsule and subacromial bursa contain melatonin receptors, which by binding to melatonin upregulates inflammatory cytokines such as interleukin-6 and increases nociception.^10^ Improving pain control can improve sleep quality, although the causes of poor sleep quality are likely multifactorial. Inability to find a comfortable sleeping posture, restrictions in range of motion, medical comorbidities, and narcotic and medication exposure can all contribute to poor sleep quality.^2^^,^^11^^,^^12^

While the causes of poor sleep are not fully understood, the effects of sleep disturbance are well-documented. Sleep dysfunction and chronic shoulder pain have been linked to psychological disorders including anxiety, depression, mood disturbances, and irritability.^6^ Poor sleep can lead to fatigue, impaired alertness and balance, and inability to perform work and daytime activities.^4^ Poor sleep quality has also been associated with impaired physiologic processes, such as anabolic states necessary for healing and maintaining bone mineral density.^4^^,^^20^

Recently, interest in sleep quality after shoulder surgery has become a focus since this has been identified as an important factor in patient recovery. Several studies of sleep quality after arthroscopic rotator cuff repair (aRCR) have shown significant sleep quality improvement, with durability out to 2 years.^7^, ^8^, ^9^^,^^11^^,^^12^ Relatively few studies have evaluated sleep response after anatomic total shoulder arthroplasty (aTSA), though the authors also found improvement in sleep quality following this intervention.^16^^,^^19^^,^^21^

To date, however, no studies have compared sleep quality response across common shoulder interventions. Our experience suggests that patients have a faster recovery of sleep quality after shoulder arthroplasty, including aTSA and reverse total shoulder arthroplasty (rTSA), compared to aRCR. Thus, we undertook a study to evaluate our patients to determine if this was the case. Our hypothesis was that sleep quality improves more rapidly after shoulder arthroplasty (aTSA and rTSA) when compared to aRCR.

## Materials and methods

### Study design

This was a prospective cohort study of consecutive patients undergoing aRCR, aTSA, or rTSA between 2018 and 2020. Institutional review board approval was obtained. Data were prospectively collected at the following predetermined time points: preoperatively, and at 2 weeks, 6 weeks, 3 months, and 6 months postoperatively. Exclusion criteria were patients less than 18 years old, non–English-speaking patients, and vulnerable patients (pregnant women, nursing home residents, employees of the institution, and those unable to give consent). All patients were enrolled in the Surgical Outcomes System database which generated surveys at predetermined time points outlined above.

### Sleep metrics and PROMs

Two sleep metrics were utilized: the Pittsburgh Sleep Quality Index (PSQI) and Visual Analog Scale–Quality of Sleep (VAS-QOS). First developed in 1989, PSQI is a composite survey of 7 components including sleep quality, sleep latency, sleep duration, sleep efficiency, sleep disturbance, the use of medication including sleeping aids, and daytime dysfunction.^5^ These 7 components generate a composite score, where increased scores reflect worse sleep and a score >5 is considered poor sleep. The PSQI was validated by Backhaus et al^3^ and has been utilized in numerous sleep quality studies within orthopedics.^7^, ^8^, ^9^^,^^11^^,^^12^ The VAS-QOS is analogous to the VAS pain score insofar as it utilizes a 100-mm visual cue as a singular metric to comprehensively assess sleep quality.^1^^,^^22^ American Shoulder and Elbow Surgeons (ASES) shoulder scores, Single Assessment Numeric Evaluation (SANE) scores, and VAS pain scores were also collected at the aforementioned time points.

### Comorbidities associated with poor sleep quality

Basic demographics were recorded including age, body mass index, gender, hand dominance, preoperative diagnosis, and a history of prior shoulder surgery. Comorbidities associated with poor sleep quality were also recorded including the Charlson Comorbidity Index, alcohol use, anxiety, depression, preoperative narcotic use, obstructive sleep apnea, and smoking history. The influence of these variables on sleep was assessed in multivariate analyses.

### Statistical methods

Univariate and multivariate regression analyses were conducted to determine the influence of comorbidities, diagnosis, and type of surgery on sleep quality. All factors with *P* value of < .1 on the univariate analysis were selected as independent variables for multivariate linear regression analyses performed for preoperative and 6-month follow-up VAS-QOS and PSQI. The distribution of procedure type was compared with chi-square analysis. Pearson bivariate correlations were calculated within sleep metrics and between sleep metrics and patient reported outcome measures (PROMs). To compare sleep metrics after arthroplasty to aRCR, the aTSA and rTSA cohorts were pooled as the Shoulder Arthroplasty cohort. *P* values of < .05 were considered statistically significant.

## Results

### Cohort description

One hundred forty-one patients who underwent shoulder surgery were included in the study (aRCR: N = 34, aTSA: N = 58, rTSA: N = 49). The most common preoperative diagnoses were glenohumeral osteoarthritis (56.7%), rotator cuff tear (24.1%), and rotator cuff tear arthropathy (14.2%). The average age was 64.1 years, and the cohort was predominantly male (56.0%) and White. For patient comorbidities, alcohol use was the most common (68.1%), followed by sleep apnea (12.8%) and depression (11.3%). Details of patient characteristics and comorbidities are included in Table I. Preoperatively, 96% of patients received a single-shot interscalene block with 20 mL 0.375% bupivacaine.

**Table I:** Patient demographics.

Sleep quality metrics	Cohort (N = 141)		RCR (N = 34)		aTSA (N = 58)		rTSA (N = 49)	
	N	%	N	%	N	%	N	%
Patient characteristics								
Age (Avg, SD)	64.1	8.9	61.0	7.0	63.0	8.7	67.4	9.3
BMI (Avg, SD)	28.1	5.1	27.5	5.2	28.7	5.0	27.6	5.2
Gender/Male	79.0	56.0	18.0	52.9	39.0	67.2	22.0	44.9
Dominant Shoulder	89.0	63.1	19.0	55.9	38.0	65.5	32.0	65.3
Prior Surgery	44.0	31.2	10.0	29.4	18.0	31.0	16.0	32.7
Comorbidities								
CCI (Ave, SD)	2.1	1.0	1.8	1.0	1.9	0.9	2.5	1.0
Alcohol	96.0	68.1	23.0	67.6	38.0	65.5	35.0	71.4
Anxiety	15.0	10.6	6.0	17.6	3.0	5.2	6.0	12.2
Depression	16.0	11.3	5.0	14.7	5.0	8.6	6.0	12.2
Preoperative Narcotics	14.0	9.9	2.0	5.9	6.0	10.3	6.0	12.2
Sleep Apnea	18.0	12.8	2.0	5.9	6.0	10.3	10.0	20.4
Smoking	5.0	3.5	1.0	2.9	1.0	1.7	3.0	6.1
Preopeative diagnosis								
RCT	34.0	24.1	34.0	100.0	0.0	0.0	0.0	0.0
OA	80.0	56.7	0.0	0.0	58.0	100.0	22.0	44.9
CTA	20.0	14.2	0.0	0.0	0.0	0.0	20.0	40.8
Fracture Sequelae	5.0	3.5	0.0	0.0	0.0	0.0	5.0	10.2
Other	2.0	1.4	0.0	0.0	0.0	0.0	2.0	4.1

*BMI*, body mass index; *RCR*, rotator cuff repair; *aTSA*, anatomic total shoulder arthroplasty; *rTSA*, reverse total shoulder arthoplasty; *CCI*, Charlson Comorbidity Index; *RCT*, rotator cuff tear; *OA*, glenohumeral osteoarthritis; *CTA*, rotator cuff tear arthropathy; *SD*, standard deviation.

### Sleep quality after arthroscopic rotator cuff repair

**Figure 1:**
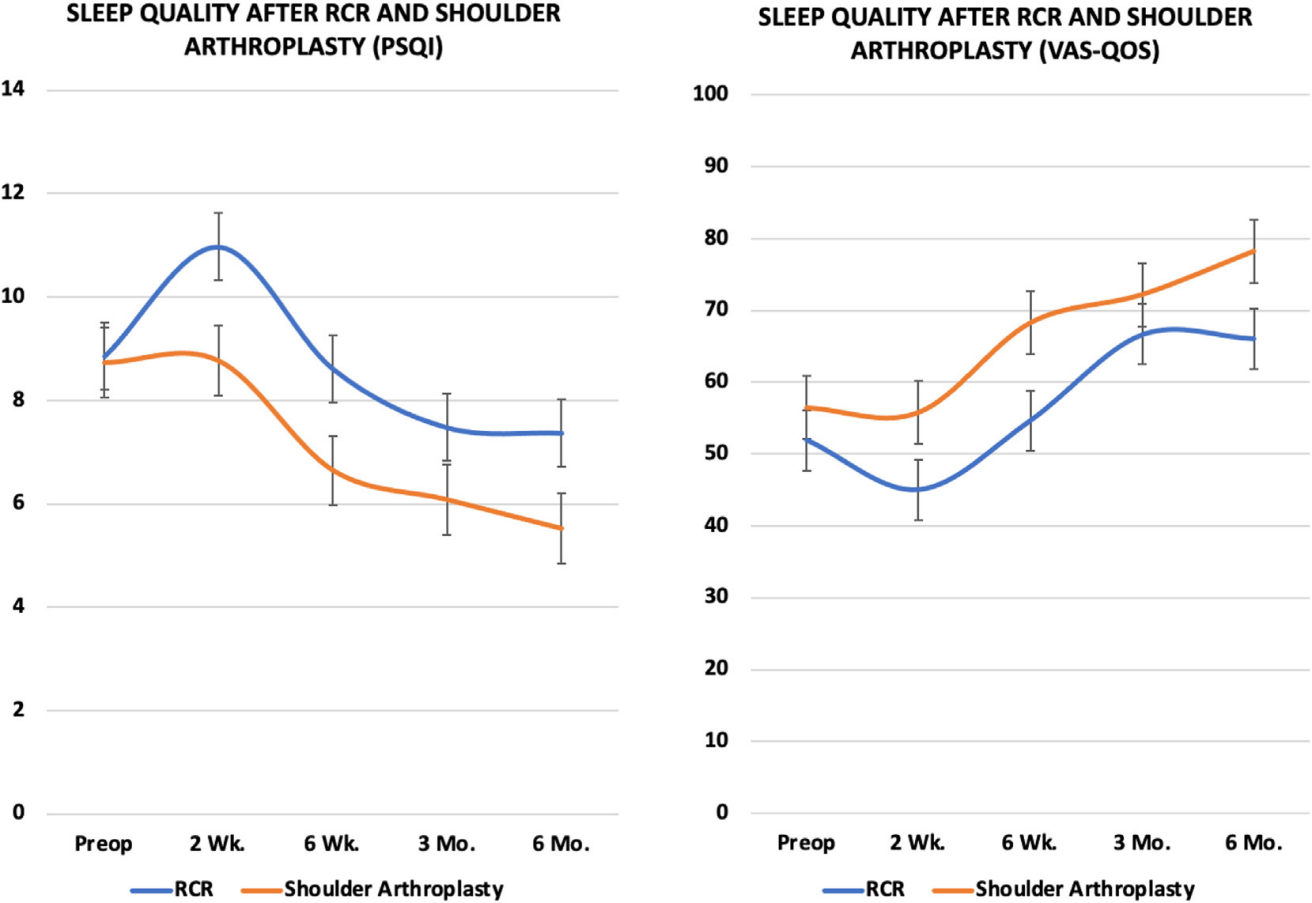
An illustration of the change in sleep quality, as measured by the PSQI and VAS-QOS, following each surgical intervention (aRCR, TSA, and rTSA). *aRCR*, arthroscopic rotator cuff repair; *RCR*, rotator cuff repair; *PSQI*, Pittsburgh Sleep Quality Index; *rTSA*, reverse total shoulder arthoplasty; *TSA*, total shoulder arthroplasty; *VAS-QOS*, Visual Analog Scale–Quality of Sleep.

In 34 patients who underwent aRCR, sleep quality as measured by both the PSQI and VAS-QOS acutely worsened at 2-week follow-up, though only the change in PSQI was statistically significant (PSQI: 8.9 vs. 11.0, *P* = .03; VAS-QOS: 51.9 vs. 45.0, *P* = .25; Fig. 1). PSQI scores trended toward improvement at subsequent visits at 6 weeks, 3 months, and 6 months postoperatively. However, there was no significant difference in PSQI score at the final follow-up compared to preoperative scores (7.4 vs. 8.9, *P* = .13). On the other hand, VAS-QOS scores improved significantly by 3 months postoperatively, and this improvement was sustained at the 6 month mark as well (Table II).

**Table II:** Sleep quality and shoulder surgery.

Surgery	PSQI					VAS-QOS				
	Score	SD	Delta	*P* value	Significance	Score	SD	Delta	*P* value	Significance
RCR										
Preoperative	8.9	3.7	-	-	-	51.9	23.4	-	-	-
Postoperative										
2 weeks	11.0	4.1	2.1	.03	∗	45.0	25.1	−6.9	.25	NS
6 weeks	8.6	4.7	−0.2	.83	NS	54.7	26.6	2.7	.69	NS
3 mo	7.5	5.0	−1.4	.30	NS	66.7	24.3	14.8	.04	∗
6 mo	7.4	4.1	−1.5	.13	NS	66.1	23.3	14.2	.02	∗
aTSA										
Preoperative	8.9	4.4	-	-	-	53.3		-	-	-
Postoperative										
2 weeks	9.2	5.0	0.3	.74	NS	54.6	24.2	1.3	.76	NS
6 weeks	6.9	4.2	−2.1	.01	∗	65.4	22.9	12.1	.01	∗
3 mo	6.4	4.2	−2.6	.01	∗	71.1	23.4	17.8	.00	†
6 mo	5.6	3.4	−3.4	.00	†	79.1	16.7	25.8	.00	†
rTSA										
Preoperative	8.5	4.4	-	-	-	60.2	21.8	-	-	-
Postoperative										
2 weeks	8.2	4.6	−0.2	.80	NS	57.1	22.6	−3.1	.49	NS
6 weeks	6.4	3.8	−2.1	.02	∗	71.8	19.5	11.6	.01	∗
3 mo	5.8	4.5	−2.7	.01	∗	73.3	21.2	21.2	.01	∗
6 mo	5.4	3.8	−3.0	.00	†	77.3	16.0	16.0	.00	∗

*RCR*, rotator cuff repair; *aTSA*, anatomic total shoulder arthroplasty; *rTSA*, reverse total shoulder arthoplasty; *PSQI*, Pittsburgh Sleep Quality Index; *NS*, nonsignificant; *VAS-QOS*, visual analog scale-quality of sleep; *SD,* standard deviation.

∗ *P* < .05.

† *P* < .01.

### Sleep quality after shoulder arthroplasty (anatomic and reverse total shoulder arthroplasty)

In the aTSA and rTSA groups, sleep quality as measured by PSQI and VAS-QOS scores improved significantly by 6 weeks, and this improvement was sustained until their most recent follow-up at 6 months (Table II). In contrast to the aRCR group, there was no significant worsening of sleep at 2 weeks postoperatively. In the aTSA cohort, the PSQI improved 3.4 points (8.9 vs. 5.6, *P* < .01) and the VAS-QOS improved 25.8 points (53.3 vs. 79.1, *P* < .01) by final follow-up. In the rTSA cohort, the PSQI improved 3.0 points (8.5 vs. 5.4, *P* < .01) and the VAS-QOS improved 17.1 points (60.2 vs. 77.3, *P* < .01) by final follow-up.

### Comparison between arthroscopic rotator cuff repair and shoulder arthroplasty (anatomic and reverse total shoulder arthroplasty)

At each time point preoperatively and postoperatively, there were no significant differences in sleep metrics between the aTSA and rTSA cohorts. There were also no significant differences in preoperative sleep quality metrics between the aRCR and the shoulder arthroplasty cohort. Shoulder arthroplasty outperformed aRCR in PSQI sleep improvement at 2 weeks and 6 months, as well as in VAS-QOS sleep improvement at all time points except 3 months (Table II).

The significance of the PSQI score was assessed by comparing with patient acceptable symptom state values published in literature. The rTSA group alone had scores surpassing the patient acceptable symptom state value of 5.5 for PSQI at 6 months of follow-up.^14^

### Poor sleepers

**Figure 2:**
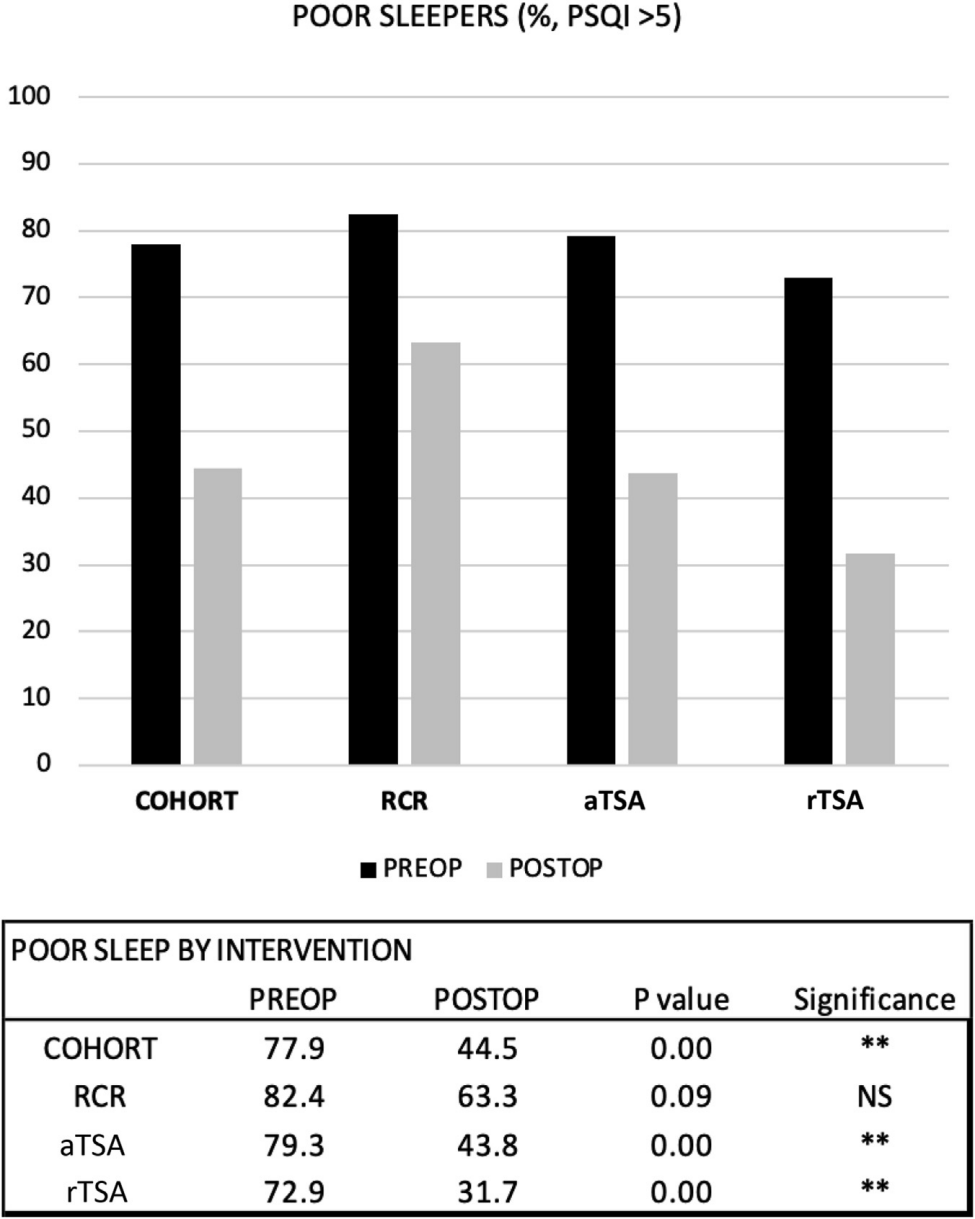
An illustration of the change in the percentage of poor sleepers (defined as PSQI >5) in each cohort (combined population, aRCR, aTSA, and rTSA) following surgical intervention. *aRCR*, arthroscopic rotator cuff repair; *RCR*, rotator cuff repair; *aTSA*, anatomic total shoulder arthroplasty; *PSQI*, Pittsburgh Sleep Quality Index; *rTSA*, reverse total shoulder arthroplasty; *NS*, not significant.

Preoperatively, 77.9% of the cohort was identified as having poor sleep (PSQI >5). 12% of the cohort took sleep-aid medications preoperatively. There was no difference in the proportion of poor sleepers preoperatively among the 3 surgical intervention groups (*P* = .56). At the final follow-up, the overall cohort in all 3 groups had significantly fewer poor sleepers (44.5%, *P* < .01). aTSA and rTSA had the greatest conversion of patients with poor sleep to normal sleep from 79.3% to 43.8% and 72.9% to 31.7%, respectively (*P* < .01; Fig. 2). aRCR trended toward a significant conversion of patients with poor sleep to normal sleep (82.4% vs. 63.3%, *P* = .09; Fig. 2).

### Multivariable regression

When controlling for patient characteristics and comorbidities, only the type of surgery had a significant influence on final VAS-QOS. Preoperative diagnosis or comorbidities did not influence sleep outcomes on multivariate analysis.

### Correlations with sleep quality metrics

PSQI and VAS-QOS scores showed a strong correlation (r = −0.74, *P* < .001). VAS pain scores had no and negligible correlations with both sleep quality metrics (PSQI and VAS-QOS, r = 0.09 and r = −0.14, respectively). There was a positive correlation between the SANE and VAS-QOS scores (R = 0.45, *P* < .001) and a positive correlation between ASES and VAS-QOS scores (R = 0.32, *P* < .01). ASES and SANE scores showed weak correlations to the PSQI (Table III).

**Table III:** Correlations of PROMs and sleep quality metrics.

Sleep quality metrics	VAS-pain		ASES		SANE	
	R	*P* value	R	*P* value	R	*P* value
PSQI	0.09	.36	−0.28	.00	−0.27	.01
VAS-QOS	−0.14	.18	0.32	.00	0.45	.00

*PROMs*, patient reported outcome measures; *ASES*, American Shoulder and Elbow Surgeons score; *SANE*, Single Assessment Numerical evaluation; *PSQI*, Pittsburgh Sleep Quality Index; *VAS-QOS*, Visual Analog Scale–Quality of Sleep.

## Discussion

The purpose of this study was to assess sleep quality response after aRCR, aTSA, and rTSA. Our main finding was that sleep disturbance is less severe in degree and shorter lasting in postoperative recovery after shoulder arthroplasty compared to aRCR. Our study shows that although the degree of overall sleep disturbance improves postoperatively in all 3 groups, the timeline of this improvement and the degree of improvement at final follow-up is different between groups. The Shoulder Arthroplasty group, which included patients who underwent either aTSA or rTSA, showed a sustained improvement in sleep quality as measured by PSQI and VAS-QOS metrics, starting at 6 weeks postoperatively until final data collection at 6 months. The aRCR group, in contrast, showed a more variable and slower course of sleep quality recovery, with acute worsening of PSQI at 2 weeks and improvement in VAS-QOS at 3 and 6 months postoperatively without any significant improvement in the PSQI scores.

Our study also found that numerous patient characteristics and comorbidities previously described to affect sleep quality have no significant association with final sleep quality scores. Lastly, correlations between PROMs and sleep quality metrics revealed that pain scores had no or negligible correlations, whereas the SANE score yielded a strong correlation to sleep quality.

Poor sleep has been recognized as one of the pathognomonic signs of shoulder pathology that influences the patients' decision to pursue treatment.^2^^,^^8^ Our study corroborated the significant prevalence of sleep disturbance, as nearly 80% of the overall cohort were designated as poor sleepers preoperatively with a PSQI score of less than 5.^3^ The rate of poor sleep in our cohort was on par with patients suffering acute orthopedic trauma (86%).^18^

Previous research has demonstrated that sleep quality improves after aRCR and shoulder arthroplasty, but no study has compared the timeline of recovery between these 2 disparate types of procedures.^2^^,^^7^^,^^8^^,^^11^^,^^16^^,^^17^^,^^19^^,^^21^ Previous studies on patients undergoing aRCR showed similar rates of improvement in sleep quality as measured by PSQI at variable time points including 4 months, 6 months, and 2 years.^2^^,^^6^^,^^11^ Studies on patients undergoing shoulder arthroplasty also showed improvements in PSQI by 6 weeks and sleep efficacy as measured by Simple Shoulder Test sleep and ASES sleep values by 3 months.^19^^,^^21^ Our data show similar findings in both the aRCR and shoulder arthroplasty groups.

Our study shows that both the VAS-QOS and PSQI have significant utility in measuring sleep quality. When compared, the VAS-QOS and PSQI had a very strong correlation (r = −0.74, *P* < .001). While the strengths of the PSQI are well-established supporting its widespread use, it is a 19-question survey which may be burdensome to patients answering several metrics. Conversely, the VAS-QOS is a single question which had a very strong correlation to the PSQI. Further, while other PROMs had negligible or no correlation to sleep metrics, the SANE score had a strong correlation to VAS-QOS scores (r = 0.45, *P* < .001), and may have utility as a convenient tool to approximate sleep metrics.

Overall, our data utiliz multiple tools for measuring sleep quality to demonstrate that recovery of sleep quality after shoulder arthroplasty is faster and more predictable than that after aRCR; however, the majority of patients undergoing these procedures should have improved sleep by 6 months after surgery. Patients should be appropriately counseled regarding expectations in changes in sleep quality postoperatively.

### Limitations

There are several limitations to this study. First, the follow-up period of 6 months may not be sufficient to illustrate the final sleep quality state following surgical intervention. Second, while the primary goal of the study was to illustrate the difference in sleep curves between shoulder arthroplasty and aRCR, the subgroup analyses of sleep quality changes, patient demographics, and surgical interventions may be underpowered. Lastly, the study does not attempt to explain what features of recovery translate to improved sleep quality, such as the use of sleep-aid medications, which was not routinely queried in patient surveys.

## Conclusion

Sleep quality improves after shoulder surgery, though the recovery curve varies by the type of surgical intervention. Sleep quality improves more rapidly following shoulder arthroplasty when compared to aRCR. The SANE score may be a useful surrogate in assessing sleep quality.

## Disclaimers:

Funding: No funding was disclosed by the authors.

Conflicts of interest: The authors, their immediate families, and any research foundations with which they are affiliated have not received any financial payments or other benefits from any commercial entity related to the subject of this article.
